# Association between Ultra-Processed Food Consumption and Metabolic Syndrome among Adults in China—Results from the China Health and Nutrition Survey

**DOI:** 10.3390/nu15030752

**Published:** 2023-02-02

**Authors:** Feng Pan, Zhihong Wang, Huijun Wang, Jiguo Zhang, Chang Su, Xiaofang Jia, Wenwen Du, Hongru Jiang, Weiyi Li, Liusen Wang, Lixin Hao, Bing Zhang, Gangqiang Ding

**Affiliations:** National Institute for Nutrition and Health, Chinese Center for Disease Control and Prevention, Beijing 100050, China

**Keywords:** ultra-processed foods, long-term consumption, metabolic syndrome, adults, China Health and Nutrition Survey

## Abstract

The prevalence of metabolic syndrome (MetS) is increasing and the relationship between ultra-processed food (UPF) consumption and MetS remains uncertain in Chinese adults. This study aimed to examine the longitudinal association of UPF consumption with the risk of MetS and its components in Chinese adults. Adults aged 18 years and above who participated in at least two waves of the China Health and Nutrition Survey (CHNS) in 2009, 2015, and 2018 were included in this analysis. Dietary intake data were collected by three consecutive 24 h dietary recalls and weighing household foods and condiments. Depending on the purpose and extent of food processing, UPFs were classified using the NOVA food classification system. A multivariate Cox proportional risk model was used to explore the association between UPF consumption (grouped by quartile: quartile 1 (Q1), quartile 2 (Q2), quartile 3 (Q3), and quartile 4 (Q4)) and risk of MetS and its components. A total of 5147 adults were included. During a median (IQR) 6.0 (3.0, 9.0) year follow-up with 31,878 person-years, 1712 MetS cases were identified, with an incidence of 33.26%. After multivariable adjustment, the risk of MetS was increased by 17% in the highest quartile with UPF consumption (HR: 1.17, 95% CI: 1.01–1.35, *p* trend: 0.047), with the lowest quartile as a reference. For the components of MetS, the risk of central obesity, raised triglycerides (TG), reduced high-density lipoprotein cholesterol (HDL-C), and raised blood pressure (BP) was increased by 33% (HR: 1.33, 95% CI: 1.18–1.51, *p* trend: <0.001), 26% (HR: 1.26, 95% CI: 1.08–1.48, *p* trend: 0.003), 25% (HR: 1.25, 95% CI: 1.07–1.46, *p* trend: 0.007), and 16% (HR: 1.16, 95% CI: 1.03–1.32, *p* trend: 0.018) in the highest quartile with UPF consumption, respectively. Adults aged 45–59 years and living in urban areas with higher UPF consumption had higher odds of MetS. These results indicate that higher long-term UPF consumption was associated with an increased risk of MetS in Chinese adults. Further studies such as intervention trials are needed to confirm the mechanism of correlation between UPF consumption and health-related outcomes. Nutritional education actions are warranted to promote a balanced diet and improve the overall dietary quality of residents to reduce the risk of MetS effectively.

## 1. Introduction

Metabolic syndrome (MetS) is defined as a cluster of cardiovascular risk factors characterized by abdominal obesity, dyslipidemia, hypertension, and high fasting blood glucose [1]. MetS has received increased attention over the past decade and has become a major public health challenge worldwide. MetS is considered to be a risk factor for multiple noncommunicable diseases (NCDs), including cardiovascular disease (CVD), stroke, coronary heart disease, type 2 diabetes (T2D), and all-cause mortality [2]. A nationally representative cross-sectional survey among Chinese adults in 2000–2001 indicated that the standardized prevalence of MetS was 9.8% [3]. About ten years later, the Chinese National Nutrition and Health Surveillance (2010–2012) reported that the prevalence of MetS in Chinese adults was 18.7% and an estimated 189 million adults living with MetS in China [4]. In 2015–2017, the standardized prevalence of MetS increased to 31.1% and nearly a third of adults had MetS in China according to the China Nutrition and Health Surveillance [5].

Previous studies have found many factors associated with MetS, including lifestyle and diet [6,7,8]. A systematic review and meta-analysis of forty observational studies reported that the “Meat/Western” dietary pattern with the characteristics of high fat, processed meat, and sweets was significantly associated with increased MetS risk [9].

NOVA is a new classification considering the nature, extent, and purpose of processing and is commonly used in recent years [10]. NOVA classifies all foods and food products into four groups: unprocessed and minimally processed foods (MPFs), processed culinary ingredients (PCIs), processed foods (PFs), and ultra-processed foods (UPFs) [11]. UPFs are industrial formulations including products made from substances extracted from foods, typically with additives, and flavorings, and commonly high in energy density, salt, added sugars, and trans fats. A growing number of studies have shown that UPF consumption is associated with an increased risk of MetS and its components [12]. Several prospective studies and randomized controlled trials have found associations between UPF consumption and increased risks of overweight/obesity [13,14,15,16]. Some prospective studies found direct significant associations between UPFs and the risk of hypertension [17,18,19]. Several European cohort studies have shown positive associations between UPF consumption and the risk of type 2 diabetes [20,21,22,23]. With regards to MetS, the cross-sectional National Health and Nutrition Examination Survey (NHANES 2009–2014) reported that for adults aged 20 years and above in the fifth quintile of UPF contribution, the risk of MetS increased by 28% (PR: 1.28, 95% CI: 1.09–1.50) compared to the contribution of the first quintile [24]. With the development of the global economy and advances in food processing technology, UPF consumption is increasing rapidly in both high-income and middle-income countries [25,26]. In China, dietary patterns are in transition from traditional dietary patterns to Western dietary patterns [27]. Li et al. showed that the mean daily UPF increased four times between 1997 and 2011 and higher UPF consumption was positively associated with overweight/obesity, diabetes, and hypertension among Chinese adults [28,29,30].

However, less research has focused on the association between UPF consumption and MetS among Chinese residents. Given the increasing prevalence of MetS and higher consumption of UPF in Chinese adults, clarification of the relationship has vital significance for the prevention of MetS through diet. Therefore, we conducted the present study to explore the association between UPF consumption and MetS and its components among Chinese adults using cohort study data from the China Health and Nutrition Survey (CHNS). The aim of our study is to derive a more precise estimation of the association between UPF consumption and MetS and provide targeted suggestions for dietary behavior in Chinese adults.

## 2. Materials and Methods

### 2.1. Study Design and Population

**Figure 1:**
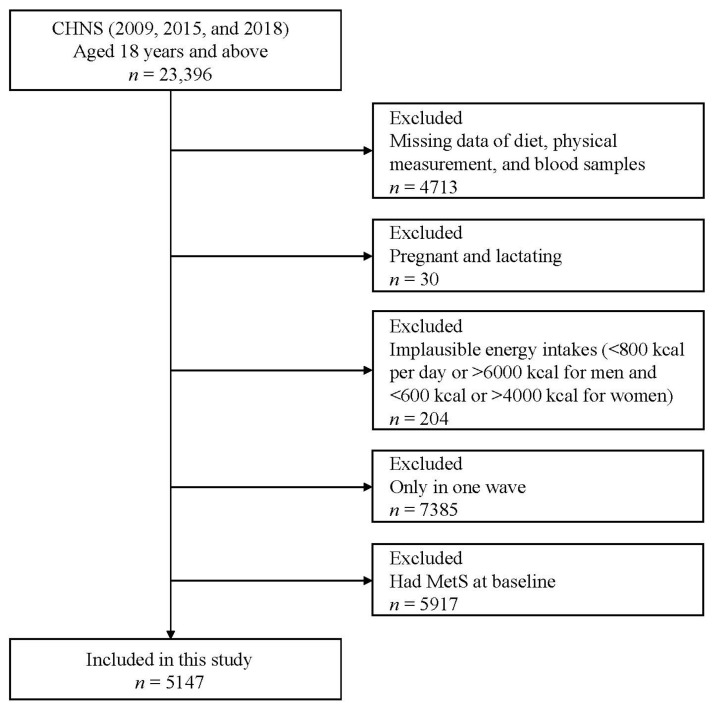
Flowchart of the participants included in this study.

The CHNS is a longitudinal, ongoing, and prospective cohort study in China which initiated in 1989 and completed 11 waves in 1991, 1993, 1997, 2000, 2004, 2006, 2009, 2011, 2015, and 2018. The CHNS used a multistage random cluster sampling method to collect the sample information including demographic geography, economic activity, community conditions, diet, and health in 15 provinces. The detailed design and procedures have been described elsewhere [31,32]. During the three waves of investigation in 2009, 2015, and 2018, blood samples were added at the same time, so the data from these three waves were used for analysis in this study. We excluded 4713 participants with deficiency of dietary, anthropometric data, and blood samples data; 30 pregnant or lactating women; 204 participants with implausible energy intakes (men: <800 kcal/day or >6000 kcal/day; women: <600 kcal/day or >4000 kcal/day); 7385 participants with only one wave; 5917 participants with MetS at baseline. Finally, a total of 5147 adults aged 18 years and above were included in this study (Figure 1).

The Institutional Review Board of the University of North Carolina at Chapel Hill and the Institutional Review Committee of the National Institute for Nutrition and Health, Chinese Center for Disease Control and Prevention, approved the survey (No. 201524, 20 August 2015). All participants provided their written, informed consent.

### 2.2. Dietary Assessment and UPF Consumption

Dietary data were collected using consecutive three-day 24 h dietary recalls (two weekdays and one weekend) for individuals in each wave of the CHNS. Meanwhile, a trained investigator weighed food items and seasonings such as oil and salt in the household inventory. Food consumption at the household level was calculated by the times of eating at home and the ratio of the energy intake of all members. Total energy and nutrients such as protein, fat, carbohydrate, and dietary sodium intake per day were calculated using the Chinese Food Composition Table [33,34].

According to the definition of NOVA classifications, food items were categorized into four groups [11]. UPF mainly includes the following food items, sugar-sweetened beverages (SSBs), packaged snacks, sweet, ice cream, chocolate, mass-produced packaged breads, cakes, desserts, biscuits, pastries, pre-prepared pies, pizza dishes, hot dogs, and sausages and other reconstituted meat products. As for uncertain food items, the presence in the list of ingredients of one or more food substances not used in kitchens including hydrolyzed proteins, “mechanically separated meat”, fructose, inverted sugar, maltodextrin, interesterified, or hydrogenated oil identified a product as UPF.

### 2.3. Definition of Metabolic Syndrome

MetS is defined using the National Cholesterol Education Program Adult Treatment Panel III (NCEP ATP III) criteria in this study. If at least three out of five of the following components were present, the person was determined to have MetS: (1) central obesity: waist circumference (WC) ≥90 cm (men) and ≥80 cm (women); (2) raised triglycerides (TG): ≥150 mg/dL or relevant specific treatment for hyperlipidemia; (3) reduced high-density lipoprotein cholesterol (HDL-C): < 1.0 mmol/L (men) and 1.3 mmol/L (women); (4) raised blood pressure: systolic blood pressure (SBP) ≥ 130 mmHg or diastolic blood pressure (DBP) ≥ 85 mmHg or specific treatment of previously diagnosed hypertension; (5) raised fasting plasma glucose (FPG): ≥ 6.0 mmol/L or diagnosed type 2 diabetes previously [35].

### 2.4. Covariates and Physical Measurement

Multiple covariates were involved in this study including gender, age, education level, geographical location, income level, smoking history, alcohol drinking status, physical activity (PA), urbanization levels, body mass index (BMI), total energy intake, dietary protein, dietary fat, dietary carbohydrate, and dietary sodium. Age was divided into three groups (18–44 years, 50–59 years, and 60 years and above). Education level was divided into two groups (junior high school or below and senior high school or above). Residence was separated into two groups (urban and rural areas). In view of the differences between the north and the south, regions were divided into the north (Beijing, Liaoning, Heilongjiang, Shandong, Henan, and Shaanxi) and the south (Shanghai, Jiangsu, Zhejiang, Hubei, Hunan, Guangxi, Chongqing, Guizhou, and Yunnan). Annual per capita household income was divided into three groups (low, medium, and high by the tertiles). Smoking history and drinking past year were divided into two groups (yes and no), respectively. Physical activities included occupational, household chores, leisure time, and transportation, and calculated into a metabolic equivalent of task (METs h/week) based on the American College of Sports Medicine Association’s recommended standard, and were then divided into three groups (low, medium, and high by the tertiles) [36]. Urbanization levels were calculated based on the economic environment of the community and the cultural and social environment and divided into three groups (low, medium, and high by the tertiles). BMI was calculated as body weight (kg) divided by the square of height (m^2^) and divided into three groups (<18.5 kg/m^2^, 18.5–23.9 kg/m^2^, and ≥24.0 kg/m^2^).

WC was measured using an inelastic flexible ruler, and weight and height were measured using an electronic weight scale and portable SECA206 stadiometer. Cholesterol oxidase-phenol and amino phenazone methods were used to measure TG and HDL-C. Blood pressure was measured using a standard mercury sphygmomanometer (Korotkoff sound). The participants were in a seated position in a quiet room for at least five minutes of rest and with the bladder emptied. The average value of three consecutive standard measurements was taken as the result for each participant. Fasting plasma glucose was measured using the hexokinase method with a Roche 702 instrument. All measurements were performed by trained professional technicians with strict quality control.

### 2.5. Statistical Analysis

Categorical and continuous variables were described by n, percentage (%) and mean, and standard deviation, respectively. Categorical and continuous variables were compared by the χ^2^ test and Kruskal–Wallis test given the skewed distribution of the data. A multivariate Cox proportional risk model was used to estimate the association between UPF consumption (grouped by quartile: quartile 1 (Q1), quartile 2 (Q2), quartile 3 (Q3), quartile 4 (Q4)) and risk of MetS and its components. We performed tests for linear trends by entering the median value of each quartile of UPF consumption as a continuous variable in the models. Meanwhile, stratified analysis was performed by covariates and interaction analysis was performed to evaluate the effect of stratification factors on the relationship between UPF consumption and the risk of MetS. All statistical analyses were conducted using SAS 9.4 (SAS Institute, Inc., Cary, NC, USA), and *p* < 0.05 (two-tailed) was defined as statistical significance.

## 3. Results

### 3.1. Baseline Characteristics

Table 1 presents the demographic and baseline characteristics of the quartile of UPF consumption. Compared with those in the bottom quartile of UPF consumption, those with higher UPF consumption were more likely to live in urban areas, have higher education levels, higher income, lower levels of physical activity, higher BMI, higher energy intake, higher protein intake, higher fat intake, higher sodium intake, and lower carbohydrate intake (*p* < 0.05). Baseline HDL-C and FPG were different between the quartile of groups (*p* < 0.05) while WC, TG, SBP, DBP as well as gender, age, smoking history, and drinking past year were not significantly different between the quartiles of UPF consumption groups (*p* > 0.05).

### 3.2. Associations of UPF Consumption with MetS and Its Components

Table 2 explores the associations of UPF consumption with MetS and its components in diverse groups. During a median (IQR) 6.0 (3.0, 9.0) year follow-up with 31,878 person-years, 1712 MetS cases were identified, with an incidence of 33.26%. After adjustment for confounding factors, such as gender, age, education level, place of residence, region, income level, smoking history, drinking status, metabolic equivalents, urbanicity, BMI, total energy, protein, fat, carbohydrate, and sodium intake, the risk of MetS was increased by 17% in the highest quartile with UPF consumption (HR: 1.17, 95% CI: 1.01–1.35, *p* trend: 0.047), with the lowest quartile as a reference.

For the associations of UPF consumption with components of MetS, after adjusting for all covariates, the risk of central obesity, raised TG, reduced HDL-C, and raised BP was increased by 33% (HR: 1.33, 95% CI: 1.18–1.51, *p* trend: <0.001), 26% (HR: 1.26, 95% CI: 1.08–1.48, *p* trend: 0.003), 25% (HR: 1.25, 95% CI: 1.07–1.46, *p* trend: 0.007), and 16% (HR: 1.16, 95% CI: 1.03–1.32, *p* trend: 0.018) in the highest quartile with UPF consumption, respectively. No correlation was observed between UPF consumption and raised FPG (HR: 1.11, 95% CI: 0.98–1.27, *p* trend: 0.141).

### 3.3. Stratified Analyses of MetS Risk and UPF Consumption

Table 3 presents the sensitivity analysis of MetS risk and UPF consumption. The results showed that the positive association of UPF consumption with risk of MetS was consistent in women, 45–59 years age group, subjects in urban areas, and southern regions. In addition, place of residence and urbanization had an interactive effect on the association between UPF consumption and risk of MetS (*p* < 0.05).

## 4. Discussion

In this study, the association between UPF consumption and MetS of Chinese adults aged 18 years and above has been evaluated through longitudinal prospective data from the CHNS. In the present study, higher UPF consumption was found to be positively correlated with MetS. The association was stronger in women, adults aged 45–59, and those living in urban areas.

The results of multiple previous studies were consistent with our findings. Lavigne-Robichaud et al. showed that comparing the lowest quintiles diet quality score, adults with the highest contribution of UPF to total daily dietary energy intake can effectively increase the risk of MetS (OR: 1.9, 95% CI: 1.14–3.17) from a 2005–2009 cross-sectional study in Canada [37]. Dana et al. also found that in adults, high consumption of UPF was associated with a higher risk for MetS (OR: 1.88, 95% CI: 1.31–2.71) and its components [38]. Previous systematic reviews and meta-analyses have shown that the highest UPF consumption was associated with a significant increase in the risk of MetS (OR: 1.79, 95% CI: 1.10–2.90) [39,40].

There are several potential plausible mechanisms that may explain the correlation between UPF consumption and MetS. Firstly, UPFs are typically high in added sugars, salt, and saturated and trans fats; excessive intake of UPFs could result in an increase in C-reactive protein (CRP) levels [41]. Moreover, further inflammatory responses may occur, and it increases the risk of MetS [42]. Secondly, higher UPF consumption is inversely associated with a poor nutritional profile and quality and deficiency intake of dietary fiber, fruit, vegetables, and legumes [43]. In addition, ingredients in UPFs such as artificial sweeteners could result in dysbiosis of gut microbiota, glucose intolerance, insulin resistance, and diverse metabolic disturbance, which then leads to the development of MetS [44,45]. Thirdly, the physical properties of food were altered by a series of industrial processes, which could result in a higher glycemic load and reduction of gut–brain satiety signaling [46,47]. The release of incretin hormones and gastric inhibitory polypeptide may increase insulin secretion and promote a greater appetite and overconsumption [48,49].

In the present study, higher UPF consumption was positively associated with the risk of central obesity (33%), raised TG (26%), reduced HDL-C (25%), and raised BP (16%), while no statistical association was found between the highest quartile group and raised FPG. An increasing body of evidence shows that UPF consumption is linked with overweight/obesity/WC [16,50,51,52]. Li et al. found that higher UPF consumption (≥50 g/d) was associated with an increased risk of overweight/obesity by 45–50% in Chinese adults aged 20 years and above using CHNS (1997–2011) [28]. The poor nutritional profile of diets with saturated fat and free sugar from UPFs contributes to more energy, weight gain, and higher odds of BMI [53]. A prospective Spanish cohort showed that adults with the highest tertile consumption of UPFs had a higher risk of developing hypertension (HR: 1.21, 95% CI: 1.06–1.37) [19]. Similarly, decreased potassium intake and increased sodium intake with UPF consumption may cause sodium/potassium imbalance, thus improving blood pressure levels [54]. In a systematic review and meta-analysis, Pagliai et al. found that no significant correlation was found between the highest UPF consumption and hyperglycemia. However, in two prospective cohort studies of UK and French adults, a diet with a higher proportion of UPFs was associated with an increased risk of type 2 diabetes (T2D) [20,21]. Of note, substances present in food packaging materials such as bisphenol-A (BPA) have been found to have endocrine-disrupting properties and a positive association with increased T2D in previous meta-analyses [55]. Collectively, higher UPF consumption has a certain adverse effect on the components of MetS. The specific mechanism and long-term health outcomes with UPF are warranted to explore.

Our findings suggest that UPF consumption was more associated with higher education levels and higher income. In Western countries, however, the situation is different. People with lower socio-economic profiles or educational levels are more likely to have higher UPF consumption [20,51]. As dietary patterns transition, UPFs are more widely available in China. People can afford to choose more food types due to the improvement in economic level while insufficient nutritional knowledge might lead them to choose durable, palatable, and ready-to-eat UPFs. We also found that adults aged 45–59 years who live in urban areas with higher UPF consumption had higher odds of MetS. With economic growth and development, China has been undergoing a rapid urbanicity and nutrition transition [56,57]. Although urbanization promotes civilization progress, it also brings about some negative consequences on health, such as low physical activity and weight gain [58]. Coincidentally, the high palatability, convenience, and easy availability of UPFs make them more accessible in urban areas and promote overconsumption. There is accumulating evidence implicating UPFs with poor dietary quality [59]. In consequence, residents in urban areas with higher UPF consumption need more attention, thereby reducing the incidence of chronic non-communicable diseases.

To the best of our knowledge, this is the first population-based prospective cohort study to examine the association between UPF consumption and the risk of MetS in Chinese adults. UPFs were classified by an updated NOVA classification system. Robust analysis was performed using three 24 h dietary recalls and data from weighing foods and condiments in household inventories. Nevertheless, there are still some limitations in this study that should be noted. First, there may be a misclassification owing to a lack of food packaging and labeling information for some uncertain food items. Second, dietary information collected by the 24 h retrospective method may lead to recall bias. Third, although potential confounding factors were adjusted, the possibility of residual confusion cannot be completely avoided. Last, the results of 24 h dietary recall may not represent long-term diet habits completely. Future studies are needed to add data from food frequency questionnaires (FFQs) and explore the correlation between UPFs and health outcomes.

## 5. Conclusions

In conclusion, this study provides prospective evidence that higher UPF consumption is positively correlated with MetS and its single component. Meanwhile, adults aged 45–59 years who live in urban areas with higher UPF consumption had a higher risk of MetS. Further studies such as intervention trials are needed to confirm the mechanism of correlation between UPF consumption and health-related outcomes. From a public health point of view, considering the gradual upward trend of UPF consumption in Chinese residents, nutrition education is warranted to promote a balanced diet and improve the overall dietary quality of residents to reduce the risk of MetS effectively.

## Figures and Tables

**Table 1:** Basic characteristics of participants by quartile of UPF consumption.

	Quartile of UPF (g/Day)				
	Q1 (<6.5)	Q2 (6.5–16.3)	Q3 (16.3–36.1)	Q4 (>36.1)	*p*-Value
Gender					0.070
Men	610 (47.4)	636 (49.4)	651 (50.6)	676 (52.5)	
Women	676 (52.6)	652 (50.6)	635 (49.4)	611 (47.5)	
Age					0.566
18–44	435 (33.8)	455 (35.3)	437 (34.0)	477 (37.1)	
45–59	511 (39.7)	501 (38.9)	525 (40.8)	483 (37.5)	
≥60	340 (26.4)	332 (25.8)	324 (25.2)	327 (25.4)	
Education level					<0.001
Junior high school or below	1050 (81.7)	984 (76.4)	954 (74.2)	777 (60.4)	
Senior high school or above	236 (18.4)	304 (23.6)	332 (25.8)	510 (39.6)	
Place of residence					<0.001
Urban areas	267 (20.8)	352 (27.3)	445 (34.6)	626 (48.6)	
Rural areas	1019 (79.2)	936 (72.7)	841 (65.4)	661 (51.4)	
Region of residence					<0.001
Northern regions	424 (33.0)	493 (38.3)	475 (36.9)	554 (43.1)	
Southern regions	862 (67.0)	795 (61.7)	811 (63.1)	733 (56.9)	
Individual annual income					<0.001
Low	536 (41.7)	493 (38.3)	411 (32.0)	303 (23.5)	
Medium	449 (34.9)	450 (34.9)	436 (33.9)	370 (28.8)	
High	301 (23.4)	345 (26.8)	439 (34.1)	614 (47.7)	
Smoking history					0.340
Yes	382 (29.7)	346 (26.9)	369 (28.7)	382 (29.7)	
No	904 (70.3)	942 (73.1)	917 (71.3)	905 (70.3)	
Drinking past year					0.549
Yes	393 (30.6)	387 (30.1)	399 (31.0)	419 (32.6)	
No	893 (69.4)	901 (70.0)	887 (69.0)	868 (67.4)	
Physical activity					<0.001
Low	424 (33.0)	405 (31.4)	422 (32.8)	464 (36.1)	
Medium	406 (31.6)	427 (33.2)	410 (31.9)	475 (36.9)	
High	456 (35.5)	456 (35.4)	454 (35.3)	348 (27.0)	
Urbanization					<0.001
Low	571 (44.4)	487 (37.8)	402 (31.3)	321 (24.9)	
Medium	414 (32.2)	424 (32.9)	422 (32.8)	417 (32.4)	
High	301 (23.4)	377 (29.3)	462 (35.9)	549 (42.7)	
BMI (kg/m^2^)					0.014
<18.5	82 (6.4)	97 (7.5)	84 (6.5)	70 (5.4)	
18.5–23.9	812 (63.1)	758 (58.9)	761 (59.2)	742 (57.7)	
≥24.0	392 (30.5)	433 (33.6)	441 (34.3)	475 (36.9)	
Energy (kcal/day)	2165.5 ± 703.2	2188.5 ± 677.1	2217.8 ± 709.9	2259.2 ± 743.4	0.025
Protein (g/day)	66.1 ± 24.1	69.9 ± 24.8	72.2 ± 26.9	76.4 ± 30.4	<0.001
Fat (g/day)	74.5 ± 38.4	79.1 ± 38.5	83.3 ± 41.3	88.8 ± 43.7	<0.001
Carbohydrate (g/day)	299.1 ± 117.2	286.6 ± 108.4	282.2 ± 106.7	278.1 ± 109.6	<0.001
Sodium (mg/day)	4380.3 ± 4188.8	4778.0 ± 3575.4	5439.8 ± 6152.9	5859.4 ± 5939.3	<0.001
WC (cm)	79.95 ± 9.33	80.35 ± 9.34	80.43 ± 10.50	80.68 ± 10.73	0.052
TG (mmol/L)	1.21 ± 0.77	1.23 ± 0.80	1.21 ± 0.83	1.18 ± 0.73	0.284
HDL-C (mmol/L)	1.49 ± 0.37	1.49 ± 0.44	1.47 ± 0.42	1.45 ± 0.35	0.045
SBP (mmHg)	121.54 ± 17.06	122.17 ± 17.09	122.47 ± 17.05	121.32 ± 15.94	0.272
DBP (mmHg)	78.58 ± 10.72	79.24 ± 10.90	79.18 ± 10.31	78.59 ± 9.75	0.199
FPG (mmol/L)	5.12 ± 1.02	5.11 ± 0.99	5.09 ± 0.90	5.03 ± 0.97	0.011

Values are given as the number of subjects, the percentage for categorical variables, and mean ± SD for continuous variables.

**Table 2:** Associations of UPF consumption with MetS and its components.

	Quartile of UPF (g/Day)				
	Q1	Q2	Q3	Q4	*p* Trend
MetS ^a^					
Median	3.3	10.9	23.5	60.8	
Model 1	1.00 (ref)	1.08 (0.95, 1.24)	1.08 (0.94, 1.24)	1.14 (0.99, 1.31)	0.126
Model 2	1.00 (ref)	1.08 (0.95, 1.24)	1.09 (0.95, 1.25)	1.16 (1.00, 1.33) *	0.075
Model 3	1.00 (ref)	1.08 (0.94, 1.23)	1.08 (0.94, 1.24)	1.17 (1.01, 1.35) *	0.047
Central obesity ^b^					
Median	3.3	11.6	26.5	67.0	
Model 1	1.00 (ref)	1.07 (0.95, 1.20)	1.12 (0.99, 1.26)	1.28 (1.13, 1.44) ***	<0.001
Model 2	1.00 (ref)	1.08 (0.96, 1.21)	1.13 (1.01, 1.28) *	1.30 (1.15, 1.47) ***	<0.001
Model 3	1.00 (ref)	1.07 (0.95, 1.20)	1.13 (1.01, 1.28) *	1.33 (1.18, 1.51) ***	<0.001
Raised TG ^c^					
Median	3.4	11.4	25.0	63.5	
Model 1	1.00 (ref)	1.06 (0.91, 1.23)	1.07 (0.92, 1.25)	1.25 (1.07, 1.46) **	0.003
Model 2	1.00 (ref)	1.06 (0.91, 1.24)	1.08 (0.92, 1.25)	1.26 (1.08, 1.48) **	0.002
Model 3	1.00 (ref)	1.06 (0.91, 1.23)	1.08 (0.92, 1.26)	1.26 (1.08, 1.48) **	0.003
Reduced HDL-C ^d^					
Median	3.4	11.0	23.7	60.3	
Model 1	1.00 (ref)	1.07 (0.92, 1.24)	1.21 (1.04, 1.40) *	1.18 (1.01, 1.38) *	0.044
Model 2	1.00 (ref)	1.08 (0.93, 1.25)	1.22 (1.05, 1.41) **	1.21 (1.04, 1.41) *	0.023
Model 3	1.00 (ref)	1.08 (0.93, 1.26)	1.24 (1.07, 1.44) **	1.25 (1.07, 1.46) **	0.007
Raised BP ^e^					
Median	3.3	11.6	26.9	66.9	
Model 1	1.00 (ref)	1.05 (0.94, 1.17)	1.05 (0.93, 1.17)	1.14 (1.02, 1.29) *	0.033
Model 2	1.00 (ref)	1.06 (0.95, 1.18)	1.05 (0.94, 1.18)	1.16 (1.03, 1.31) *	0.022
Model 3	1.00 (ref)	1.05 (0.94, 1.18)	1.04 (0.93, 1.16)	1.16 (1.03, 1.32) *	0.018
Raised FPG ^f^					
Median	3.4	11.3	25.2	64.4	
Model 1	1.00 (ref)	1.04 (0.92, 1.18)	1.04 (0.92, 1.17)	1.07 (0.94, 1.22)	0.404
Model 2	1.00 (ref)	1.06 (0.94, 1.20)	1.05 (0.93, 1.19)	1.09 (0.96, 1.24)	0.287
Model 3	1.00 (ref)	1.05 (0.93, 1.19)	1.07 (0.94, 1.21)	1.11 (0.98, 1.27)	0.141

^a^ n = 5147. ^b^ n = 5558. ^c^ n = 5412. ^d^ n = 5411. ^e^ n = 5695. ^f^ n = 5585. * *p* < 0.05, ** *p* < 0.01, *** *p* < 0.001. Model 1 adjusted gender, age, education level, place of residence, regions, and income level; Model 2 further adjusted smoking history, drinking status, metabolic equivalents, and urbanicity based on Model 1; Model 3 further adjusted BMI, total energy intake, protein intake, fat intake, carbohydrate intake, and sodium intake based on Model 2.

**Table 3:** Stratified analyses of MetS risk and UPF consumption.

	Quartile of UPF (g/Day)				
	Q1 (<6.5)	Q2 (6.5–16.3)	Q3 (16.3–36.1)	Q4 (>36.1)	*p* for Interaction
Gender					0.208
Men	1.00 (ref)	1.12 (0.93, 1.36)	1.06 (0.87, 1.28)	1.09 (0.89, 1.33)	
Women	1.00 (ref)	1.00 (0.82, 1.22)	1.10 (0.90, 1.34)	1.26 (1.02, 1.55) *	
Age					0.093
18–44	1.00 (ref)	0.98 (0.76, 1.26)	0.91 (0.70, 1.18)	0.97 (0.74, 1.27)	
45–59	1.00 (ref)	1.16 (0.94, 1.44)	1.27 (0.91, 1.40)	1.29 (1.03, 1.61) *	
≥60	1.00 (ref)	1.02 (0.80, 1.32)	1.16 (0.90, 1.50)	1.27 (0.96, 1.66)	
Education level					0.250
Junior high school or below	1.00 (ref)	1.14 (0.98, 1.32)	1.14 (0.97, 1.33)	1.13 (0.95, 1.34)	
Senior high school or above	1.00 (ref)	0.83 (0.60, 1.14)	0.88 (0.64, 1.20)	1.13 (0.84, 1.51)	
Place of residence					0.013
Urban areas	1.00 (ref)	1.19 (0.89, 1.60)	1.11 (0.84, 1.47)	1.41 (1.07, 1.86) *	
Rural areas	1.00 (ref)	1.05 (0.90, 1.22)	1.09 (0.92, 1.28)	1.01 (0.84, 1.21)	
Region of residence					0.365
Northern regions	1.00 (ref)	0.95 (0.76, 1.18)	1.00 (0.80, 1.25)	1.02 (0.81, 1.28)	
Southern regions	1.00 (ref)	1.17 (0.98, 1.39)	1.14 (0.95, 1.36)	1.29 (1.06, 1.55) **	
Individual annual income					0.252
Low	1.00 (ref)	1.04 (0.84, 1.29)	1.05 (0.83, 1.32)	1.22 (0.95, 1.58)	
Medium	1.00 (ref)	1.17 (0.94, 1.47)	1.06 (0.83, 1.34)	1.05 (0.81, 1.36)	
High	1.00 (ref)	0.98 (0.74, 1.30)	1.11 (0.85, 1.44)	1.17 (0.90, 1.51)	
Urbanization					0.008
Low	1.00 (ref)	1.13 (0.91, 1.39)	1.09 (0.87, 1.36)	0.99 (0.77, 1.29)	
Medium	1.00 (ref)	1.01 (0.79, 1.29)	1.02 (0.79, 1.32)	0.96 (0.74, 1.24)	
High	1.00 (ref)	1.08 (0.82, 1.41)	1.15 (0.86, 1.49)	1.45 (1.11, 1.89)	

* *p* < 0.05, ** *p* < 0.01.

## Data Availability

Data sharing is not applicable to this study according to the National Institute for Nutrition and Health, Chinese Center for Disease Control and Prevention.
